# CCL2 release by airway smooth muscle is increased in asthma and promotes fibrocyte migration

**DOI:** 10.1111/all.12444

**Published:** 2014-06-16

**Authors:** S R Singh, A Sutcliffe, D Kaur, S Gupta, D Desai, R Saunders, C E Brightling

**Affiliations:** 1 Institute for Lung Health, Department of Infection, Immunity and Inflammation, University of LeicesterLeicester, UK

**Keywords:** airway smooth muscle, asthma, chemokine (C-C motif) ligand 2, chemotaxis, fibrocyte

## Abstract

**Background:**

Asthma is characterized by variable airflow obstruction, airway inflammation, airway hyper-responsiveness and airway remodelling. Airway smooth muscle (ASM) hyperplasia is a feature of airway remodelling and contributes to bronchial wall thickening. We sought to investigate the expression levels of chemokines in primary cultures of ASM cells from asthmatics *vs* healthy controls and to assess whether differentially expressed chemokines (i) promote fibrocyte (FC) migration towards ASM and (ii) are increased in blood from subjects with asthma and in sputum samples from those asthmatics with bronchial wall thickening.

**Methods:**

Chemokine concentrations released by primary ASM were measured by MesoScale Discovery platform. The chemokine most highly expressed by ASM from asthmatics compared with healthy controls was confirmed by ELISA, and expression of its cognate chemokine receptor by FCs was examined by immunofluorescence and flow cytometry. The role of this chemokine in FC migration towards ASM was investigated by chemotaxis assays.

**Results:**

Chemokine (C-C motif) ligand 2 (CCL2) levels were increased in primary ASM supernatants from asthmatics compared with healthy controls. CCR2 was expressed on FCs. Fibrocytes migrated towards recombinant CCL2 and ASM supernatants. These effects were inhibited by CCL2 neutralization. CCL2 levels were increased in blood from asthmatics compared with healthy controls, and sputum CCL2 was increased in asthmatics with bronchial wall thickening.

**Conclusions:**

Airway smooth muscle-derived CCL2 mediates FC migration and potentially contributes to the development of ASM hyperplasia in asthma.

Asthma affects over 300 million people worldwide and its prevalence is ever increasing. It still remains a significant cause of morbidity and mortality 1. Asthma is a complex heterogeneous disease characterized by variable airflow obstruction, airway inflammation, airway hyper-responsiveness and airway remodelling 2.

Airway remodelling denotes structural changes in the airway wall including epithelial denudation and shedding, basement membrane thickening, goblet cell hyperplasia, subepithelial fibrosis, mucus hypersecretion and increased airway smooth muscle (ASM) mass as a consequence of ASM hyperplasia and hypertrophy 2. Although the cause of ASM hyperplasia is not yet completely understood, several theories have been proposed. These include epithelial–mesenchymal transition, ASM proliferation and/or survival and migration of resident mesenchymal stem cells or peripheral blood progenitors [fibrocytes (FCs)] to the ASM bundle and their differentiation into ASM 3,4. Several chemotactic pathways driving FC recruitment to ASM have been suggested including chemokines and growth factors 5–7. However, in our earlier work, we reported that platelet-derived growth factor (PDGF) was an important chemotactic factor for FC recruitment but only contributed to about a fifth of FC migration towards ASM 5. Furthermore, we were unable to support a role for CCR3, 7, CXCR3 or 4 in mediating migration towards the ASM 5. Therefore, other important mechanisms promoting FC recruitment to the ASM bundle in asthma need to be identified.

We hypothesized that in asthma, there is an increased release of chemotactic factors from ASM that promote FC recruitment and that the concentration of this mediator or mediators in the airway is related to bronchial wall thickening. We provide evidence for CCR2 expression on human FCs and a role for CCL2 in modulating FC migration towards the ASM bundle in asthma.

## Materials and methods

### Subjects

Healthy control and asthmatic subjects were recruited from respiratory clinics and hospital staff and by means of local advertising. Healthy subjects had no history of respiratory disease. Subjects underwent extensive evaluation including an extensive history, skin prick tests for common aeroallergens, spirometry, methacholine challenge tests, sputum induction 8 and Asthma Control Questionnaire (ACQ6) 9. Some subjects also underwent video-assisted bronchoscopy with bronchial biopsies and/or thoracic computerized tomography (CT) scan as previously described 10,11. Qualitative assessment of bronchial wall thickening was performed as previously described by a single radiologist 11.

The diagnosis of asthma was made by a respiratory physician based on history and one or more of the following objective criteria (maximum diurnal peak expiratory flow variability >20% over a 2-week period, significant bronchodilator (BD) reversibility defined as an increase in FEV_1_ of >200 ml post-BD or a PC_20_FEV_1_ methacholine of <8 mg/ml). Severe asthma was defined in accordance with the American Thoracic Society (ATS) workshop on refractory asthma 12. Informed consent was obtained from all subjects, and the study was approved by the Leicestershire, Northamptonshire and Rutland Research Ethics Committee.

### Cell isolation and culture

Pure ASM bundles were isolated from biopsies obtained at bronchoscopy from well-characterized asthmatics and healthy volunteers with additional nonasthmatic samples obtained from lung resection. The clinical characteristics of ASM donors are as shown in Table1. ASM cells were cultured in DMEM with Glutamax-1 supplemented with 10% FBS, 100 U/ml penicillin, 100 μg/ml streptomycin, 0.25 μg/ml amphotericin, 100 μM nonessential amino acids and 1 mM sodium pyruvate. ASM cell characteristics were determined by flow cytometry with α-smooth muscle actin (SMA)-fluorescein isothiocyanate (FITC)-conjugated antibody (Sigma, Gillingham, Dorset, UK) 13.

**Table 1:** Clinical characteristics of airway smooth muscle donors

	Nonasthmatic (*n* = 18)	Asthmatic (*n* = 18)
Age in years*	55 (4)	48 (3)
Male, *n* (%)	7 (39)	11 (61)
Smoking, *n* (%)	10 (56)	8 (44)
Current	05	06
Ex	05	02
Smoking (pack years)*	16 (5)	2 (1)
FEV_1_ current*	2.9 (0.3)	2.7 (0.2)
FEV_1_% predicted *	87.4 (4.4)	79.9 (4.9)
FVC*	3.7 (0.3)	4.0 (0.2)
FEV_1_/FVC ratio*	78.9 (6.8)	65.9 (2.8)

* Data expressed as mean (SEM) unless otherwise stated.

Fibrocytes were isolated from peripheral blood and cultured in fibronectin-coated (40 μg/ml) T25 tissue culture flasks, as described previously 5. After 24 h, nonadherent cells were removed. After 7–14 days, adherent cells were washed with Hanks’ balanced salt solution and harvested with Accutase (eBioscience, Wembley, UK). Cell counts and viability of the initial isolated peripheral blood mononuclear cell fraction and the final adherent cells were determined using trypan blue stain. Viability was consistently >95%. In parallel, PBMC preparations were seeded onto fibronectin-coated eight-well chamber slides (2 × 10^5^ cells per well) and cultured as above. Fibrocyte purity and differentiation were assessed by immunofluorescent staining for CD34, αSMA and collagen I, as described previously 5. Fibrocyte purity was routinely >95%, with morphologically distinct T cells accounting for the contaminants.

### Analysis of basal mediator release in human ASM supernatants

Airway smooth muscle cells were seeded in T75 culture flasks at a density of 1.7 × 10^5^ and grown to confluence over 2 weeks. Airway smooth muscle cells were serum-deprived in insulin/transferrin/sodium selenite (ITS) media (Sigma) for 24 h. Fresh ITS media were then added, and cell-free supernatants were collected after 24 h by centrifuging at 1300 rpm for 8 min at 4°C. These were then stored at −20°C until required. Basal levels of mediators were analysed using both multiplex and single enzyme-linked immunosorbent assay (ELISA) kits. Supernatants analysed for chemokine levels were corrected for ASM cell numbers (10^6^ cells). We measured CCL2-5, CCL11, CCL13, CXCL8, CXCL10 and CXCL11 [MesoScale Discovery (MSD); MesoScale Diagnostics, LLC (Rockville, MD, USA)] in pooled ASM supernatants of healthy (*n* = 11) and asthmatic (*n* = 10). Limits of detection were 0.24–10 000 pg/ml. ELISA kits were used to examine CCL2 (R&D Systems Inc., Abingdon, UK) in healthy and asthmatic ASM supernatants from individual donors. Limit of detection for CCL2 was 15.6–1000 pg/ml.

### Measurement of CCL2 in supernatants from FC/ASM co-cultures and monocultures

Airway smooth muscle cells were seeded onto 10 μg/ml fibronectin-coated 60-mm^2^ tissue culture dishes at a density of 2 × 10^5^ to reach confluence after 24 h. Airway smooth muscle cells were serum-starved overnight. Fibrocytes were harvested with accutase and labelled with CFSE (1 : 1000 in PBS), prior to seeding at a density of 1 × 10^5^ onto ASM cultures or 10 μg/ml fibronectin-coated culture dishes as parallel FC monocultures. Airway smooth muscle monocultures were also maintained in parallel. Cell-free supernatants for ELISA were collected following a further 7–8 days, spun at 1300 rpm for 8 min at 4°C and stored at −20°C until required. CCL2 levels were then analysed by ELISA (R&D Systems Inc.).

### Analysis of CCR2 receptor expression

Fibrocytes were stained with mouse monoclonal anti-human CCR2 antibody (R & D Systems Europe Ltd., Abingdon, UK) or isotype control (clone IgG2b; R & D Systems Europe Ltd.) indirectly labelled with FITC (Dako, Ely, Cambridgeshire, UK) and assessed by flow cytometry (BD FACScan; BD, Oxford, UK) as previously described 14 and immunofluorescence following counterstaining of cell nuclei with 4′,6′-diamidino-2 phenylindole (Sigma) as described previously 15.

### Fibrocyte migration

Migration of adherent FCs was studied using a previously validated chemotaxis assay 16,17. Airway smooth muscle cells (2.5 × 10^5^/well) were seeded onto fibronectin-coated (1 μg/ml) eight-rectangular-well plates and allowed to adhere overnight before removal by scraping of half the ASM below a predrawn line on the well underside. Fibrocytes (4–8 × 10^4^/well) were then added for 72 h before experimentation, with pertussis toxin (PTx) (0.5 μg/ml, from Sigma) added 18 h prior to experimentation. Photographs of the FCs were taken along a predrawn line 5 mm below the edge of the multicell layer or equivalent position in control wells at 1.5-h intervals over a 4.5-h time course. The migration of individual FCs was tracked manually, and the distance of migration was obtained by a blinded observer.

Additionally, we used a 24-well transwell migration assay to measure the migration of detached differentiated FCs that had been in culture for 1 week after isolation. The transwell inserts were coated with 40 μg/ml fibronectin for 1 h at 37°C. Recombinant human CCL2 (50 ng/ml or 100 ng/ml; R & D Systems Europe Ltd.) or conditioned medium from ASM cultures that had been activated for 24 h with TNF-α (10 ng/ml) was added to the bottom compartment of each well in the presence or absence of mouse monoclonal anti-human CCL2 antibody (5 μg/ml; R & D Systems Europe Ltd.) or isotype control and with the exception of the negative controls. Fibrocyte cultures were thoroughly washed several times with fresh medium to remove nonadherent cells. Cultures were then detached using accutase (eBioscience), and 1 × 10^5^ FCs were added to the top chamber of each well in duplicate. Cells were incubated for 4 h at 37°C, at which point the cells present in the lower well were recovered by means of pipetting, centrifuged at 250 ***g*** for 5 min and then resuspended in PBS for counting on a flow cytometer by a blinded observer. Fibrocytes were readily distinguishable from T cells by their forward and side scatter characteristics on flow cytometry.

### Measurement of CCL2 in blood and sputum

CCL2 levels in plasma collected from healthy control subjects and patients with asthma were measured (MSD; MesoScale Diagnostics, LLC). Sputum CCL2 levels were measured using multiplex assays from Myraid RBM (Austin, TX, USA) as described previously, and some of these subjects had participated in an earlier study 18.

### Statistics

Statistical analysis was performed using PRISM version 6 (GraphPad, La Jolla, CA, USA). Analysis between groups was performed by one-sample, paired or unpaired *t-*tests or Mann–Whitney tests for parametric and nonparametric data, respectively. For sputum CCL2 measurements, subjects were dichotomized into presence or absence of bronchial wall thickening. Between-group differences were analysed by unpaired *t*-tests or Fisher's exact test. A value of *P* < 0.05 was considered significant.

## Results

### ASM primes FC chemokinesis through a G-protein-coupled receptor

**Figure 1:**
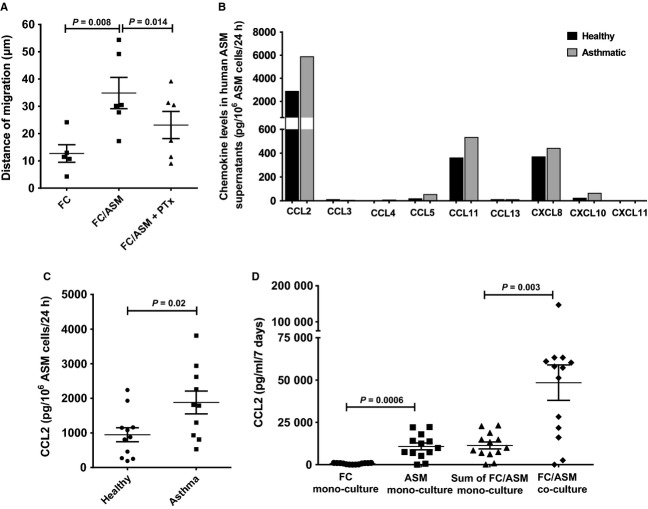
Chemokine (C-C motif) ligand 2 (CCL2) levels are increased in asthmatic airway smooth muscle (ASM) supernatants. (A) ASM primes migration of fibrocytes (FCs) through a G-protein-coupled receptor as demonstrated by inhibition with pertussis toxin (PTx); (B) basal chemokine levels in pooled healthy and asthmatic supernatants of ASM cells assessed by MesoScale Discovery platform (MSD); CCL2 levels in (C) healthy and asthmatic ASM cell supernatants and (D) supernatants from FCs and ASM mono- and co-cultures assessed by ELISA. Data expressed as mean ± SEM.

We first explored the potential role of G-protein-coupled receptors in regulating ASM-mediated FC chemokinesis. The distance of migration of adherent elongated FCs was a mean of 12.7 ± 3.2 μm from the point of origin over 4.5 h (*n* = 5). Airway smooth muscle significantly potentiated chemokinesis of FCs by over twofold [34.9 ± 5.7 μm, *n* = 6, *P* = 0.008, (Fig.1A)] from the point of origin over 4.5 h. PTx, an inhibitor of Gαi receptor – G-protein coupling, significantly attenuated ASM-primed FC chemokinesis by 34 ± 9%, [23.2 ± 5.0 μm from the point of origin, *n* = 6, *P* = 0.014 (Fig.1A)].

### CCL2 levels are increased in ASM supernatants from subjects with asthma

The role of a G-protein-coupled receptor in mediating FC migration towards ASM implicated the involvement of chemokines. Therefore, to determine whether the basal release of chemokines is differentially expressed by ASM from asthmatic subjects *vs* healthy controls, the concentration of several key chemokines was initially screened in pooled samples from healthy (*n* = 11) and asthmatic ASM supernatants (*n* = 10) using the MSD platform. The greatest difference observed in chemokine concentration between ASM supernatants from asthmatics and healthy controls was a twofold increase in basal CCL2 levels (Fig.1B). CCL2 concentrations in these supernatants from individual subjects were measured by ELISA and confirmed that CCL2 was significantly increased in ASM supernatants from asthmatics compared with healthy controls (1880 ± 329 *vs* 947 ± 203 pg/10^6^ cells; *P* = 0.02, Fig.1C).

We then assessed CCL2 release in supernatants from FCs and ASM in co-culture and the sum total of CCL2 levels in supernatants from the corresponding FC and ASM monocultures. Airway smooth muscle monocultures showed significantly higher CCL2 release compared with FC monocultures (*n* = 13, *P* = 0.0006). CCL2 levels were found to be significantly increased in FC/ASM co-cultures (48472 ± 10508 pg/ml, *n* = 13, *P* = 0.003) relative to the sum total of corresponding FC/ASM monocultures [11372 ± 2064 pg/ml (Fig.1D)]. In co-cultures, ASM and FC proliferation was not increased compared with the sum of their respective monocultures over 7 days (8.7 ± 14.7% increase in FC/ASM in co-culture *vs* sum of FCs and ASM in monoculture, *P* = 0.57). There was also no difference in the ratio of ASM/FCs in monoculture *vs* co-culture (28.1 ± 5.9 *vs* 22.9 ± 5, respectively, *P* = 0.35).

### CCR2 is expressed on peripheral blood human FCs

**Figure 2:**
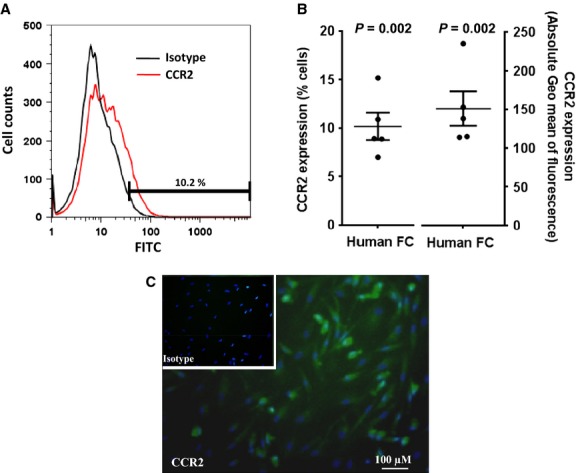
CC chemokine receptor (CCR)2 is expressed on peripheral blood fibrocytes (FCs). (A) A representative histogram (*n* = 5 independent experiments) illustrating cell surface CCR2 expression; (B) proportion of FCs expressing CCR2 with absolute geometric mean of CCR2 fluorescence on human FCs relative to its isotype assessed by flow cytometry; (C) CCR2 expression on FCs confirmed by immunofluorescence. Data expressed as mean ± SEM.

CCL2 affects cell function through binding to cell surface CCR2 receptors. To investigate the role of CCL2 in modulating FC migration, we therefore first assessed CCR2 expression on peripheral blood FCs. An example histogram of CCR2 expression demonstrated by flow cytometry is shown in (Fig. 2A). The proportion of primary cultured peripheral blood FCs that expressed CCR2 on their cell surface was 10 ± 1 (6–14)% [*n* = 5 (Fig.2B)]. These observations were consistent with a significantly higher geometric mean for CCR2 fluorescence relative to its relevant isotype control antibody [Δ geometric mean 151 ± 22, *n* = 5 (Fig.2B)]. Qualitative CCR2 immunoreactivity was also evident by immunofluorescence in cultured FCs [representative image from *n* = 5 (Fig.2C)].

### CCL2 promotes migration of peripheral blood FCs

**Figure 3:**
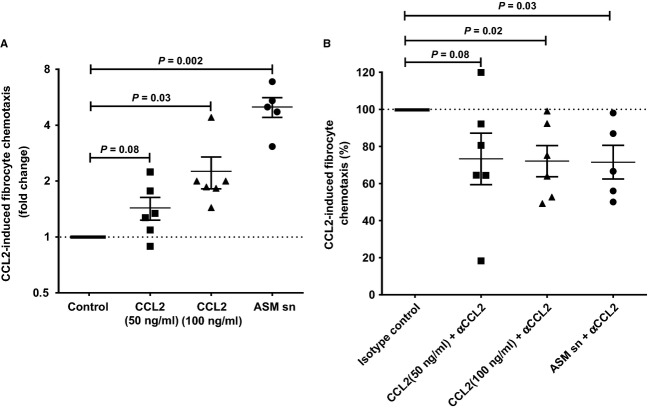
Chemokine (C-C motif) ligand 2 (CCL2) regulates human fibrocyte (FC) migration. (A) Effect of recombinant CCL2 and airway smooth muscle (ASM) supernatant (sn) on FC chemotaxis (log_2_ scale) compared with their relevant control; (B) effect of CCL2 neutralizing antibody (αCCL2) on recombinant CCL2- and ASM sn-induced FC chemotaxis (%) compared with their relevant isotype control. Data expressed as mean ± SEM.

We next sought to understand the role of CCL2 on FC migration. Differentiated FCs migrated significantly in response to stimulation with 100 ng/ml of recombinant CCL2 [2.3 ± 0.4-fold greater than control medium, *n* = 6, *P* = 0.03 (Fig.3A)] and ASM supernatant stimulated with TNF-α [5.0 ± 0.6-fold greater than control medium, *n* = 5, *P* = 0.002 (Fig.3A)]. CCL2 (50 ng/ml) failed to induce a significant increase in migration of differentiated human FCs [1.4 ± 0.2-fold greater than control medium, *n* = 6, *P* = 0.08 (Fig.3A)]. CCL2 neutralizing antibody significantly inhibited recombinant CCL2-induced (100 ng/ml) migration of FCs by 28.0 ± 8.4%, *n* = 6, *P* = 0.02 (Fig.3B) and ASM supernatant-induced migration by 28.5 ± 9.1%, *n* = 5, *P* = 0.03 (Fig.3B) relative to relevant isotype controls.

### *In vivo* CCL2 measurements

**Figure 4:**
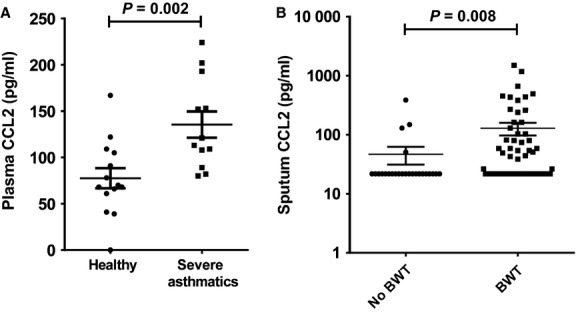
Chemokine (C-C motif) ligand 2 (CCL2) levels are increased in asthmatics and are associated with bronchial wall thickening. (A) Measurement of plasma CCL2 from healthy controls and asthmatics; (B) sputum CCL2 levels are increased in asthmatics with bronchial wall thickening (BWT) compared to those without. Data expressed as mean ± SEM.

To validate and confirm our *in vitro* observations, we measured CCL2 levels *in vivo*. Plasma CCL2 concentration was significantly increased in severe asthmatics (*n* = 14) compared with healthy controls (*n* = 12) [136 ± 14 *vs* 78 ± 11 pg/ml, *P* = 0.002 (Fig.4A)]. Sputum CCL2 concentrations were significantly increased in asthmatics with bronchial wall thickening (129 ± 31 pg/ml, *n* = 67, *P* = 0.008) compared to those with no evidence of bronchial wall thickening [47 ± 16 pg/ml, *n* = 25 (Fig.4B)]. The clinical characteristics of the subjects with or without bronchial wall thickening are shown in Table2. In those with bronchial wall thickening *vs* those without, there were no differences in demographics, lung function, atopy, total IgE or sputum differential cell counts between asthmatic subjects with or without bronchial wall thickening.

**Table 2:** Clinical characteristics of subjects recruited for sputum CCL2 analysis

	Bronchial wall thickening (*n* = 67)	No bronchial wall thickening (*n* = 25)	*P*-value
Age in years*	49 (2)	50 (3)	0.98
Male, *n* (%)	32 (48)	11 (44)	0.81
Smoking, *n* (%)	28 (42)	12 (48)	0.64
Current	04	04	
Ex	24	08	
Smoking (pack years)*	8.2 (2.3)	4.5 (2.1)	0.37
Oral corticosteroid use, *n* (%)	31 (46)	8 (32)	0.24
Daily prednisolone dose (mg)*	4.6 (0.7)	4.1 (1.4)	0.74
ICS dose†	2000 (1600–2000)	2000 (1000–2000)	0.87
Pre-BD FEV_1_*	2.2 (0.1)	2.3 (0.2)	0.57
Post-BD FEV*	2.3 (0.1)	2.5 (0.2)	0.55
Pre-BD FEV_1_% predicted*	72.5 (2.9)	75.6 (4.1)	0.57
Post-BD FEV_1_% predicted*	78.0 (2.9)	79.9 (4.2)	0.73
Pre-BD FEV_1_/FVC ratio*	68.4 (1.6)	71.4 (2.7)	0.33
Post-BD FEV1/FVC ratio*	70.6 (1.5)	73.7 (2.5)	0.30
Atopy present, *n* (%)	34 (51)	9 (36)	0.24
Asthma severity classification			
GINA 3	02	01	
GINA 4	34	16	
GINA 5	31	08	
Sputum neutrophils, %‡	69.3 (60.8–74.3)	71 (58.3–91.8)	0.57
Sputum macrophages, %‡	14.9 (11.3–21.5)	8.8 (3.8–28.5)	0.92
Sputum eosinophils, %‡	3.9 (2.5–6.0)	2.3 (0.5–5.8)	0.76
Sputum lymphocytes, %‡	0.67 (0.5–1.0)	0.71 (0.3–1.3)	0.96
Sputum epithelium, %‡	1.5 (0.8–2.0)	0.9 (0.3–2.5)	0.44
Total IgE, IU/l*	523 (99)	202 (45)	0.06
Sputum CCL2 (pg/ml)*	129 (31)	47 (16)	0.008
BMI, kg/m^2^*	29.6 (0.7)	30.1 (1.4)	0.74
ACQ6 score*	2.3 (0.1)	2.2 (0.2)	0.80

BD, bronchodilator; ACQ6, Asthma Control Questionnaire; BMI, body mass index; GINA, global initiative for asthma; CCL2, chemokine (C-C motif) ligand 2.

Data expressed as

* mean ±SEM

‡ median (interquartile range)

† Doses of all inhaled corticosteroids were converted to the equivalent dose of beclomethasone dipropionate and expressed here as median dose (interquartile range)

## Discussion

We report here for the first time that constitutive release of CCL2 by primary ASM is increased in asthma, that ASM-derived CCL2 promotes migration of FCs and that increased sputum CCL2 is associated with bronchial wall thickening. Together, these findings implicate a role for CCL2 in FC trafficking to the ASM in asthma and possibly to consequent ASM hyperplasia and airway remodelling.

Fibrocytes are peripheral blood-derived mesenchymal cell progenitors, and their numbers are increased in ASM bundles and in the peripheral blood of asthmatics 5. This increase correlates with the degree of airflow obstruction 3, and they have been implicated in contributing to ASM hyperplasia. However, there is a limited understanding of the mechanisms regulating their trafficking to the lungs. Earlier studies have implicated a role for CXCR4/CXCL12 axis in the recruitment of peripheral blood progenitors to the lung 6. Interestingly, ASM-derived PDGF contributes to one-fifth of FC chemokinesis towards ASM bundles 5. This suggested that there are other potential mechanisms involved in this process that need further elucidation. We have previously shown that ASM-mediated FC chemokinesis was not modulated by activation of CCR3, CXCR3 and CXCR4 by their cognate ligands, and even though the CCL19/CCR7 axis played a critical role in recruitment of differentiated mesenchymal cells within ASM bundles, it did not have any predominant effect in the recruitment of progenitors 5,16. However, here we found that ASM primes chemokinetic migration of FCs predominantly via a Gi/o G-protein-dependent mechanism, implicating a potential role of alternative chemokines in driving FC recruitment to the ASM bundle that we had hitherto overlooked.

We confirm here the role of recombinant CCL2 in mediating FC migration 19 and demonstrate for the first time that ASM-derived CCL2 promotes chemotaxis of FCs. Our findings are consistent with a body of evidence supporting a role for CCL2 in asthma. Airway structural and inflammatory cells have been identified to be important sources for CCL2 20–22. CCL2 levels are higher in asthmatic bronchoalveolar lavage (BAL) fluid, and an allergen challenge induces a further significant release of CCL2 in BAL fluid of patients with asthma 23. Histological findings demonstrate CCL2 expression in the bronchial epithelium, subepithelial macrophages, blood vessels and ASM of asthmatic and nonasthmatic bronchial biopsies 24. Comparatively, the expression is stronger in asthmatic epithelium and subepithelial layer 24. These observations are consistent with *in vivo* findings. Increased CCL2 levels are observed in animal models of allergic asthma 25. Blocking CCL2 with a neutralizing antibody inhibits release of macrophage-derived inflammatory mediators including leukotriene B4 (LTB4), prostaglandin E2 (PGE2) and thromboxane B2 (TXB2) and recruitment of monocytes, T cells and eosinophils to the lung along with a reduction in the degree of bronchoconstriction 26. Here, we extend these earlier observations by demonstrating that the constitutive release of CCL2 by ASM from asthmatics is increased compared with healthy controls. We further demonstrate increased CCL2 levels in FC/ASM co-culture beyond the sum of the concentration of CCL2 released from each cell type in monoculture. Various studies have shown an increased chemokine production in co-cultures 27,28. However, the mechanism remains to be determined yet. We also observed an increase in CCL2 in blood from asthmatic subjects consistent with a previous observation made by Chan and colleagues who recorded higher serum CCL2 levels in asthmatics which were further increased during an acute asthma attack 29. For the first time, we also report that sputum CCL2 concentration is increased in asthmatics with bronchial wall thickening.

Bronchial wall thickening is the hallmark of airway remodelling, which together with airway inflammation contributes to airway hyper-responsiveness and/or airflow obstruction 30,31. The major determinant of bronchial wall thickening, luminal narrowing and airflow obstruction is increased ASM mass 31–33. Thoracic CT scans are widely recognized as a useful noninvasive measure of airway remodelling. Thicker airway walls have been identified in severe asthmatics relative to mild asthmatics or healthy subjects, and this correlates with the degree of airflow obstruction 11,34. Our recent work extended these observations by identifying distinct asthma phenotypes using CT-derived airway indices of airway remodelling and air trapping 35. Our finding here that increased sputum CCL2 levels are associated with bronchial wall thickening in asthmatics implicates CCL2 in airway remodelling and is consistent with the view that CCL2 might promote ASM hyperplasia and consequent changes in airway geometry. Interestingly, in an earlier study, we did not find an association between sputum CCL2 and airway wall geometry 18. However, in this study, we only measured the apical segment of the right upper lobe in fewer subjects than studied in the current study; therefore, the differences we found here might be a consequence of the inclusion of all visible airways or that the previous study was underpowered. These observations underscore the need for further studies to define the relationship between CCL2 expression in the bronchial mucosa, bronchial secretions and airway remodelling.

One of the main limitations of this study is the cross-sectional design. Future studies need to include longitudinal follow-up as this would allow a better understanding of the dynamic association between CCL2 release and the natural history of airway remodelling. Another limitation is subjectivity in the assessment of bronchial wall thickening due to the use of qualitative methods to describe these changes. However, the observations were made by a single observer with excellent intrasubject variability and allowed the assessment of all the visible airways. Unfortunately, not all the CTs used in this study were captured using a standardized algorithm required for quantitative assessment of the whole airway tree 35; and therefore, this approach should be considered for future studies. In this study, we did not measure CCL2 release from healthy and asthmatic ASM cells following priming with pro-inflammatory cytokines, which would otherwise play a key role in disease pathogenesis *in vivo*. It has been previously reported that CCL2 expression is up-regulated by IL-1, TNF-α and endothelin-1 36–38. It is therefore possible that we may have underestimated the expression and significance of asthmatic ASM-derived CCL2 and its potential contribution to FC migration. Based on our previous and current observations, ASM-derived PDGF 5 and CCL2 significantly contribute to FC chemotaxis. Other ASM-derived mediators are likely to play a minor role in FC migration. Another criticism of this study could be the limited effects of CCL2 neutralizing antibody in blocking the effects of recombinant CCL2 on FC chemotaxis. A more potent neutralizing antibody would provide a definitive understanding of the proportionate role of CCL2 in promoting *in vitro* FC migration. Despite these limitations, we believe that our observations are robust and if at all, possibly underestimate the role of CCL2 in driving FC migration.

In conclusion, these observations confirm previous findings of increased CCL2 levels in asthma and suggest that ASM-derived CCL2-mediated activation of CCR2 promotes FC migration. Increased levels of sputum CCL2 are associated with bronchial wall thickening. Targeting CCL2-mediated FC trafficking to the airway may provide novel therapies to modulate ASM hyperplasia and airway remodelling.

## Abbreviations

ASM: airway smooth muscle
CCL2: chemokine (C-C motif) ligand 2
CT: computerized tomography
GINA: global initiative for asthma
LTB4: leukotriene B4
PDGF: platelet-derived growth factor
PGE2: prostaglandin E2
PTx: pertussis toxin
TXB2: thromboxane B2
